# Understanding genetic variation in *in vivo* tolerance to artesunate: implications for treatment efficacy and resistance monitoring

**DOI:** 10.1111/eva.12194

**Published:** 2014-08-11

**Authors:** Laura C Pollitt, Derek Sim, Rahel Salathé, Andrew F Read

**Affiliations:** 1 Center for Infectious Disease Dynamics, Department of Biology, Pennsylvania State UniversityUniversity Park, PA, USA; 2 Centre for Immunity, Infection and Evolution, University of EdinburghEdinburgh, UK; 3 Department of Entomology, The Pennsylvania State UniversityUniversity Park, PA, USA; 4 Fogarty International Center, National Institutes of HealthBethesda, MD, USA

**Keywords:** artemisinin, clearance rates, drug resistance, genetic variation, malaria, *Plasmodium chabaudi*

## Abstract

Artemisinin-based drugs are the front-line weapon in the treatment of human malaria cases, but there is concern that recent reports of slow clearing infections may signal developing resistance to treatment. In the absence of molecular markers for resistance, current efforts to monitor drug efficacy are based on the rate at which parasites are cleared from infections. However, some knowledge of the standing variation in parasite susceptibility is needed to identify a meaningful increase in infection half-life. Here, we show that five previously unexposed genotypes of the rodent malaria parasite *Plasmodium chabaudi* differ substantially in their *in vivo* response to treatment. Slower clearance rates were not linked to parasite virulence or growth rate, going against the suggestion that drug treatment will drive the evolution of virulence in this system. The level of variation observed here in a relatively small number of genotypes suggests existing ‘resistant’ parasites could be present in the population and therefore, increased parasite clearance rates could represent selection on pre-existing variation rather than *de novo* resistance events. This has implications for resistance monitoring as susceptibility may depend on evolved traits unrelated to drug exposure.

## Introduction

The evolution of drug resistance in pathogens is a major public health concern, threatening many of the health advances achieved in the last century. Further to the direct impacts for individual health through treatment failure, drug resistance has considerable economic costs, including the development of new drugs and research into alternative treatments (French 2005). Malaria parasites provide a classic case study for the problems of resistance, as the introduction of new drugs has inevitably been followed by the evolution and spread of resistant mutants, resulting in many previously successful drugs becoming ineffectual (White 2004; World Health Organization 2010a,b). Artemisinin-based drugs (hereafter, artemisinins) are the current front-line drugs against malaria parasites and are highly valued for their ability to rapidly clear infections (White 2004, 2008). However, in the last few years, reports from western Cambodia have shown that some infections are being cleared more slowly by treatment, raising concerns of developing resistance (Noedl et al. 2008; Dondorp et al. 2009; Amaratunga et al. 2012).

Artemisinin resistance is however controversial (Meshnick 2012; White 2012; Dondorp and Ringwald 2013; Ferreira et al. 2013; Krishna and Kremsner 2013). Although slower clearance rates have been reported, treatment failures are rare (but see Carrara et al. 2013) and molecular markers or known mechanisms for resistance are lacking (Dondorp et al. 2010; Anderson et al. 2011). Monitoring of drug efficacy therefore relies on calculating the parasite clearance curve in treated infections, with increased infection half-life (time for parasite density to reduce by 50%) taken as a sign of resistance (White 2011). This can be problematic, as the speed of parasite clearance from an infection will depend on a combination of host and parasite factors (Sowunmi et al. 2010; Stepniewska et al. 2010). For example, even in untreated infections, parasite numbers will decrease after an initial peak due to host immune killing and depletion of red blood cell resources, factors which will vary both over the course of individual infections and between infections in different hosts (Mideo et al. 2011; Fairlie-Clarke et al. 2013). Additionally, parasite strains show significant variation in many of the traits underlying replication and survival within the host (Ferguson et al. 2003; Bell et al. 2006; Pollitt et al. 2011). Variation in these traits (e.g. intrinsic replication rate) is also likely to lead to standing variation in initial susceptibility to drug treatment and the ability of any surviving parasites to re-establish an infection post-treatment. The potential for standing genetic variation in susceptibility to modern antimicrobial drugs has been demonstrated in bacteria, where strains from ancient arctic ice-cores have mutations conferring resistance to modern antibiotics (Perron et al. 2014).

Characterizing intrinsic variation in drug susceptibility for malaria parasites (i.e. variation existing on first exposure rather than present after drug-driven evolution) is important because, as with many pathogens, there is a delay in between reports of decreased drug efficacy and the discovery of a mechanism or marker for resistance. Therefore, monitoring for resistance relies on measuring infection half-life (White 2011), and to accurately detect a new resistance mutant, it is necessary to quantify how much variation is present prior to drug-imposed selection. Additionally, if intrinsically low susceptibility correlates with other parasite traits, treatment-imposed selection may result in an increase in other clinically important traits such as virulence to the host (Schneider et al. 2008, 2012).

Characterizing the variation in treatment susceptibility among genotypes of *Plasmodium falciparum* (the most clinically important human malaria parasite) is challenging as mixed strain infections are common (Juliano et al. 2010), and it is difficult to control other infection variables, not least because untreated controls are not possible for ethical reasons. This makes it challenging to disentangle the relative contribution of the parasite and host factors which generate the substantial variation observed in the infection clearance rates of treated infections in humans (Cheeseman et al. 2012). Susceptibility of *P. falciparum* parasite lines can be tested *in vitro*, and substantial genetic variation has been shown in this context (Beez et al. 2011), but it remains unclear how this relates to susceptibility and infection dynamics in the host. Here, we use five previously unexposed genotypes of the rodent malaria parasite *Plasmodium chabaudi* to provide the first *in vivo* test for variation in susceptibility of naïve malaria parasites to artemisinins. In addition to testing for standing variation in susceptibility, we test for correlations between susceptibility and other parasite life-history traits, including virulence, which could be inadvertently selected for by drug pressure (Schneider et al. 2008, 2012).

## Methods

### Parasites, hosts and infections

The *P. chabaudi* genotypes used in this study were originally collected from thicket rats (*Thamnomys rutilans*) in the Congo (Carter 1978), maintained as part of the WHO Registry of Standard Malaria Parasites (The University of Edinburgh), transported to Penn State University and stored in liquid nitrogen. Four of the genotypes (ER, AJ, AQ, and AS) were wild-type isolates which had never been exposed to drug pressure, and a fifth was a pyrimethamine-resistant line (AS_8p(pyr-1A)_) derived from an AS ancestor after exposure to a high dose of pyrimethamine (Walliker et al. 1975). In what follows, we denote these two AS lines as AS (wt) and AS (pry R), respectively. None of the five lines had previously been exposed to artemisinin-based drugs.

For each parasite genotype, infections were initiated in 10 female C57Bl/6 (6–10 week old) mice via intraperitoneal (IP) injection of 10^6^ parasites, with half the mice receiving drug treatment and half acting as an untreated control (total number of mice = 50). All mice received 0.05% PABA-supplemented drinking water to enhance parasite growth (Jacobs 1964), were fed on Laboratory Rodent Diet 5001 (LabDiet; PMI Nutrition International, Brentwood, MO, USA) and kept on a 12:12 L:D cycle. Drug treatment was given as an IP injection of artesunate (Sigma-Aldrich, Gillingham, Dorset, UK) dissolved in sterile water and administered twice daily for 5 days [at ∼11 am and 4 pm on days 6–10 post-infection (PI)] at a dose of 8 mg/kg.

Experimental infections were monitored daily from day 3 to 24 PI and on day 26. During sampling, 5 μL of blood was taken from each host to estimate total parasite densities by quantitative PCR (see Huijben et al. 2011). One mouse (AJ drug treated) had substantially lower than expected parasite densities in the days before treatment, as this was most likely due to experimental error in infection, this mouse was excluded from further analysis.

### Infection dynamics

Infection clearance rate during drug treatment was calculated by fitting the slope of the linear decline in parasite density over time (on a natural log scale) during the period of drug treatment (day 6–11 PI) (Stepniewska et al. 2010; Flegg et al. 2011; White 2011). For some infections (3/25 mice), there was a lag before clearance, during which the density of parasites continued to increase for 1 day after treatment but then declined. This lag is also seen in some human infections (White 2011). For these cases, the slope was fitted from day 7 rather than day 6 (Flegg et al. 2011). Additionally, some infections had fallen below the detection threshold by day 11. This will also bias the clearance rate calculation (White 2011); therefore, data until day 10 were used for these infections (3/25 mice). A linear model provided a good fit for our data (mean *R*^2^ = 0.94 ±0.01 SE), and clearance slopes were used to individually calculate the infection half-life during drug treatment for each infection [half-life in hours = (natural log of 2/absolute clearance slope) × 24]. As we took parasite density measurements every 24 h (once per cell cycle), it is possible the peak parasite density could have occurred between measurements. To ensure that our choice of start point for the clearance curves did not alter our findings we re-fitted curves with a fixed start point on day 6 (the start of treatment). This led to a slightly worse fit to the data (mean *R*^2^ = 0.91 ± 0.02 SE) but did not affect the conclusions of our analysis (see Supporting Information).

Drug treatment was given at the peak of infection, as this corresponds to the onset of symptoms, and therefore about the point at which treatment is generally sought in human infections. At this point, untreated infections will also decline in density, due to a combination of resource limitation and host immunity (Mideo et al. 2008). To determine the relative contribution of drug action and the deteriorating within-host environment on clearance rates, we also fitted clearance curves to parasite densities between days 6 and 11 for control infections (mean *R*^2^ = 0.89 ± 0.01 SE) and used these slopes to calculate infection half-life in the absence of treatment.

Intrinsic growth rates were calculated for all infections by fitting the slope of the linear increase in log_10_ parasite density between days 3 and 6 PI. A linear model provided a good fit for our data (mean *R*^2^ = 0.96 ± 0.004 SE) showing that our infections were growing exponentially during this period of the infection.

### Virulence to the host

Across the whole infection period, daily measurements were taken for mouse weight and red blood cell density (by Flow Cytometry, Beckman Coulter Counter, High Wycombe, UK; see Ferguson et al. 2003), which are common indicators of health in this system (de Roode et al. 2005; Bell et al. 2006; Pollitt et al. 2014; Spence et al. 2013). To account for any initial variation between mice, these measurements were subtracted from the value on the first day of monitoring for each infection to give a daily measurement of the change in red blood cell density (anaemia) and weight.

### Statistical analysis

All statistical tests were carried out using R version 2.14.1 (http://www.R-project.org). Infection half-lives (log-transformed) and the cumulative density in the week post-treatment (area under the curve) were analysed using general linear models (Gaussian error structure). Infection dynamics over time were analysed using linear mixed-effect models with mouse fitted as a random effect. As is common with repeated-measure parasite data, there was significant temporal autocorrelation within our dataset (*P* > 0.0001 in all analyses across multiple days; see Supporting Information). We therefore fitted a corAR1 autocorrelation structure with mouse nested within day into our models (Zuur et al. 2011; Pollitt et al. 2012). For all analyses over multiple days of infection, day was included as a factor to account for nonlinear infection dynamics over time. We followed model simplification by sequentially dropping the least significant term and comparing the change in deviance with and without the term to chi-squared distributions, until the minimal adequate model was reached. Degrees of freedom correspond to the difference in the number of terms in the model in relation to the residual degrees of freedom. Detailed tables for all our statistical models can be found in the Supporting Information.

## Results

### Line and treatment effects on parasite dynamics

**Figure 1:**
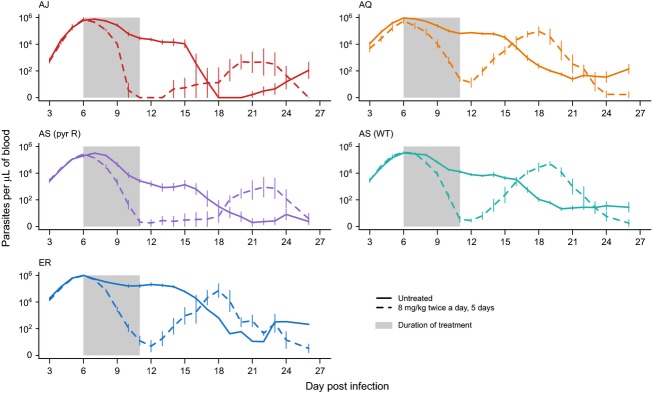
Infection dynamics in treated and untreated infections. Lines show the mean from four or five independent infections of each of five parasite clones (denoted AJ, AQ, AS (pry R), AS (wt) and ER). Error bars show the standard error of the mean. In drug-treated infections, mice received 8 mg/kg artesunate twice a day on days 6–10 PI. Area of grey shading indicates the period of drug treatment.

Over the entire period of monitoring, infection dynamics depended on both the parasite genotype and the interaction between genotype and treatment (parasite ID × drug × day: 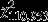 = 744.2, *P* < 0.001; Fig.1; Table S1.1). Therefore, we split our data to examine, for treated infections, (i) parasite clearance during treatment, and (ii) parasite recrudescence post-treatment.

### Infection clearance

**Figure 2:**
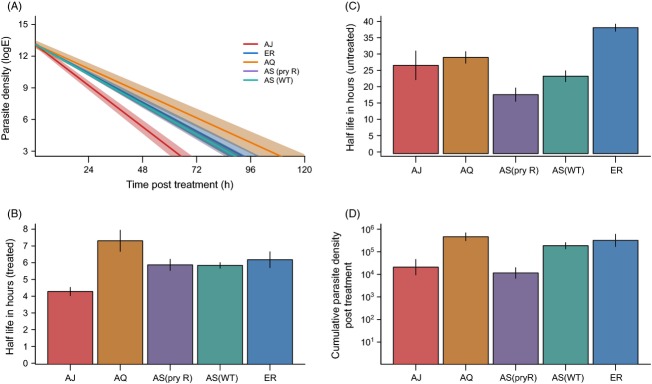
Genetic variation in the response to drug treatment, deteriorating within-host environment and post-treatment recrudescence. (A) Parasite clearance curves for treated infections over the period of drug treatment controlling for density at the time of first drug dose. Lines show the mean clearance curve across four or five replicate infections and the shaded area shows the standard error. (B) Clearance rates from A transformed into an infection half-life. (C) Infection half-life in untreated infections for days 6–11 postinfection. (D) Mean cumulative parasite densities in treated infections between the end of drug treatment and day 26. Bars show the means of four or five infections, and bars show the standard error of the means.

In drug-treated infections, clearance rates significantly correlated with parasite density at the onset of treatment, and this effect was independent of parasite line (parasite half-life ∼ density at day 6 PI; 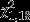 = 5.58, *P* = 0.03); infections with a higher density cleared at a faster rate and this was controlled for in subsequent analyses. In addition clearance, rates differed significantly between parasite lines (Fig.2B) with the least susceptible parasite genotype (AQ; mean = 7.31 h) having a half-life of over 3 h (1.71-fold increase) more than the most susceptible (AJ; mean = 4.28 h) (Fig.2B; parasite half-life ∼ parasite line; 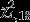 = 10.75, *P* < 0.0005; Table S1.2). A Tukey *post hoc* test revealed that the two AS lines and ER had similar half-lives under drug treatment, but AQ had a significantly longer half-life than all other lines, and AJ had a significantly shorter half-life (Table S1.2). Extrapolating clearance curves to the predicted time to full clearance showed that infections with ER would take 58 h (2.4 days) longer to clear than infections with AJ (a 1.7-fold increase).

To examine what might be driving strain differences in clearance rates in treated infections, we removed parasite genotype from the models and tested for correlations with other infection parameters. There was a significant negative relationship between infection half-life and intrinsic growth rate; infections which had grown at a faster rate pretreatment were cleared by drugs more rapidly (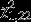 = 15.55, *P* < 0.001). There was, however, no significant effect of a delay in clearance (parasite density falling only after the 2nd day of treatment) (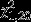 =0.77, *P* = 0.39) or red blood cell density at either the start or end of treatment (day 6 RBC 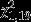 = 0.43, *P* = 0.52; day 11 RBC 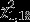 =0.001, *P* = 0.99; Table S1.3).

As in treated infections, parasite clearance in untreated mice (defined as the rate of decline in parasite numbers between day 6 and day 11 PI, see Methods) significantly correlated with peak infection density (parasite density at day 6 PI; 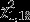 = 5.11, *P* = 0.036), and there was significant variation between parasite lines (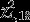 = 6.48, *P* < 0.005; Fig.2C; Table S1.4). Importantly, when we analysed half-life across all infections (treated and untreated), there was a highly significant interaction between the effect of drug treatment and parasite genotype (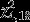 = 9.10, *P* < 0.0001; compare Fig.2B,C; Table S1.5), as well as significant negative correlations with pre-peak growth rate and parasite density at peak (which were independent of differences between parasite lines) (intrinsic growth rate 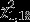 = 4.15, *P* = 0.049; density at peak 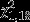 = 9.48, *P* < 0.005; Table S1.5). There was no significant correlation with red blood cell density at the peak of infection (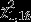 = 0.04, *P* = 0.85). Therefore, parasite lines varied in their response to declining within-host conditions, their susceptibility to drug treatment, and how these factors interact to reduce density in treated infections.

### Recrudescence post-treatment

Infection dynamics post-treatment (day 12–26 PI) were significantly affected by parasite genotype, with the pyrimethamine-resistant AS line and the AJ line recrudescing later and reaching a lower density than the other lines (parasite ID × day: 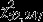 = 160.5, *P* < 0.0001; Fig.1). None of the other factors included in the model explained any additional variation in the parasite dynamics post-treatment (lag in response to treatment: 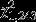 = 0.004, *P* = 0.95; intrinsic growth rate pre-treatment: 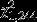 = 1.18, *P* = 0.68; parasite half-life: 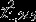 = 0.13, *P* = 0.72; parasite density at end of treatment: 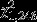 = 1.44, *P* = 0.23; Table S1.6). A similar picture emerged when we compared the total parasite biomass produced after drug treatment (area under the curve for each treated infection between day 12 and day 26), where there were significant differences between the parasite lines (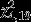 = 9.74, *P* < 0.0005; Fig.2D; see Table S1.7 for pairwise comparisons). Again, the wild-type AS line reached significantly higher densities post-treatment than the pyrimethamine-resistant AS line [Tukey *post hoc* test AS (wt) versus AS (pyr R) *z* = 3.75, *P* < 0.005].

As with our analysis of clearance rates, we examined the parasite traits that may be driving strain differences in recrudescence by fitting the models again, but omitting strain as a factor. Across all infections, there was no significant relationship between infection growth rates before treatment and cumulative density post-treatment (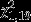 = 0.35, *P* = 0.77). Neither was there any significant relationship with parasite half-life during treatment (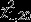 = 0.29, *P* = 0.60) nor red blood cell density at the end of treatment (day 11 RBC 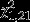 = 2.95, *P* = 0.10). There was a significant positive relationship between the density of parasites remaining at the end of drug treatment and the cumulative density post-treatment (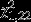 = 7.5, *P* = 0.012; Table S1.8), but this effect was not independent of parasite genotype (Table S1.7).

### Line and treatment effects on infection virulence

**Figure 3:**
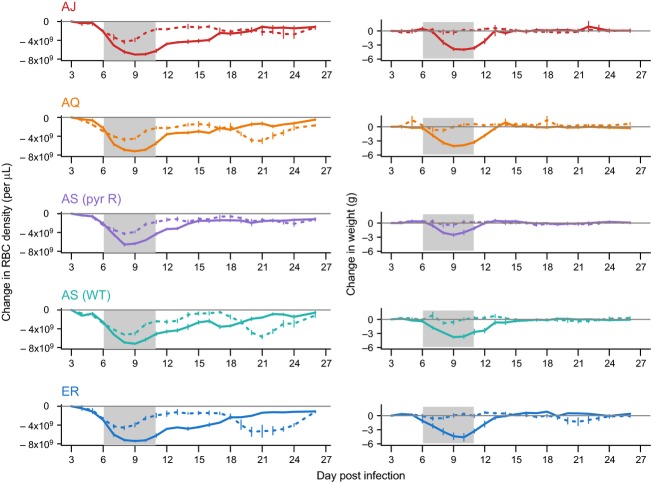
Parasite genotype and drug treatment effect on host health. Values are calculated loss or gain in red blood cell density (left panels) and weight (right panels) relative to values on day 3 post-infection. Dashed lines show treated infections, and solid lines show untreated. The grey line is a reference for no change, and bars show the standard error of the mean from four or five replicate infections per genotype and treatment. The grey-shaded area indicates the period of drug treatment.

Over the whole infection, drug treatment substantially reduced anaemia and weight loss (change in RBC density: 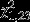 = 5.41, *P* = 0.02; change in mouse weight: 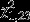 = 18.1, *P* < 0.0001; Fig.3; Table S1.9). Parasite genotype had a borderline effect on anaemia (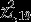 = 9.18, *P* = 0.057) but no effect on weight loss (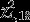 = 3.06, *P* = 0.54). There was no significant interaction between treatment and parasite genotype on either measures of virulence (change in RBC density: 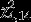 = 2.90, *P* = 0.58; change in mouse weight: 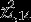 = 3.66, *P* = 0.45). However, as infection dynamics and virulence varied substantially over the course of the infection, we split our analysis to examine weight loss and anaemia over two time periods, from the start of treatment to day 17 PI (corresponding to the initial period of acute infection), and from day 18 to day 26 PI (corresponding to the period of recrudescence in those infections where it occurred).

During the initial acute infection period, there was a significant interaction between parasite genotype and drug treatment for anaemia (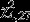 = 16.78, *P* < 0.005; Fig.3; Table S1.10); treatment led to the greatest reduction in anaemia relative to the untreated controls for infections with AJ and ER and the smallest effect for the two AS genotypes. During this same period, drug treatment led to significantly less weight loss across all infections (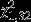 = 27.38, *P* < 0.0001), but there was no additional effect of parasite genotype (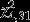 = 5.10, *P* = 0.28) or an interaction between genotype and treatment (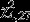 = 4.00, *P* = 0.41) (Fig.3; Table S1.10).

Treatment during the initial acute infection significantly increased anaemia later in the infection, and this depended on which parasite genotype the mouse was infected with (drug treatment × parasite genotype: 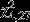 = 11.07, *P* = 0.026; Fig.3; Table S1.11). Mice which had received treatment became more anaemic late in the infection, and this was most pronounced in infections with ER, AQ and AS (wt); the genotypes which had the greatest recrudescence during this period. The interaction between drug treatment and parasite genotype also had a significant effect on weight loss (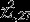 = 10.12, *P* = 0.038; Table S1.11), however, the effect sizes were small and no mice experienced large changes in weight during the late infection period (Fig.3).

## Discussion

Here, we demonstrate, for the first time *in vivo*, that malaria parasites have substantial standing variation in their susceptibility to moderate artesunate treatment, despite no previous exposure. While this study focuses on rodent malaria parasites as a model system, *P. chabaudi* has been shown to evolve a resistance phenotype in response to selection with artemisinin, similar to the resistance phenotype reported in human infections (Pollitt et al. 2014). The difference between infection half-life during drug treatment of the most and least susceptible genotype examined here was more than 3 h (Fig.2B), equating to a time to clearance difference of 2.4 days (the least susceptible genotype took 1.7 times longer to clear than the most susceptible). Considering the relatively small number of genotypes in our study, this variation is striking. In human malaria parasites, the difference in mean half-life between parasite infections reported as resistant or sensitive is around 4 h (Cheeseman et al. 2012), but increases of 1 and 3 h have also been taken as evidence of changing susceptibility to artemisinins (Amaratunga et al. 2012; Cheeseman et al. 2012; Phyo et al. 2012; Das et al. 2013; Kyaw et al. 2013) and an 1.75 times increase in time to clearance of as evidence for resistance (Dondorp et al. 2009). Therefore, if variation in human malaria parasites was similar to in our rodent model, selection on existing standing variation could be sufficient to explain the changes seen in response to artemisinins. This is in keeping with the finding that highly resistant *P. falciparum* parasite lines showed decreased diversity consistent with a selective sweep but not complete genetic separation from susceptible lines (Cheeseman et al. 2012). Additionally, our data suggest that variation in parasites traits other than mutations directly interfering with drug molecule action (traditionally considered resistance alleles, such as those encoding target site modifications, efflux pumps and so on) may be important in treatment efficacy, making the search for a molecular marker of resistance more complex.

Parasite genotypes also varied significantly in their ability to recrudesce following treatment. This had significant implications for disease severity, with those genotypes which had the earliest and largest recrudesces causing anaemia in their hosts comparable or greater than the original treated infection [AS (wt), AQ & ER in Figs2D and 3]. Presumably greater recrudesces would also provide a fitness advantage to the parasite by providing greater opportunity to transmit (Pollitt et al. 2014). Drug treatment itself caused more severe anaemia later in infections (Fig.3). We assume this is because untreated infections are better seen by the immune system early in infections and consequently better controlled later on.

Intriguingly, the pyrimethamine-resistant AS genotype had significantly depressed growth in the week post-treatment compared to its wild-type ancestor even though both genotypes were cleared by drug treatment at the same rate. Whether reaching a lower density post-treatment is due to a cost of resistance to another drug or to unrelated mutations cannot be determined from this comparison of two lines, but would be an interesting hypothesis to test. If parasites resistant to a previous antimalarial were more susceptible to new drugs, this could provide an opportunity for resistance management via drug cycling or combination therapy (Bonhoeffer et al. 1997; White 2004).

In untreated infections, there was substantial variation between genotypes in the rate at which parasite numbers decreased after the peak of infection. The magnitude of the additional clearance through drug action in treated infections also significantly varied between parasite lines, but not in a way that could be predicted from the dynamics in untreated infections (there was a highly significant interaction between treatment and parasite genotype). Therefore, parasite lines varied in their response to declining within-host conditions, their susceptibility to drug treatment and how these factors interact to reduce density in treated infections. This is in keeping with the recent finding that the clearance rate of parasites selected for drug resistance depend on the within-host conditions (Pollitt et al. 2014). Infections with the highest growth rates in the initial infection (intrinsic growth rate), and therefore the greatest number of parasites at the peak of infection, were most rapidly cleared in both treated and untreated hosts. There was no evidence that this was due to resource limitation; there was no significant relationship between parasite half-life and red blood cell density. Possible explanations are faster growing parasites could be more vulnerable to artesunate treatment, and/or faster growing parasites may trigger a stronger immune response. How drug action and immune killing combine to determine treatment efficacy is an important question for future work.

Previous research in rodent malaria has suggested that drug pressure could select for increased virulence because treatment was more efficacious against low virulence parasites (Schneider et al. 2008, 2012). Our results did not support this hypothesis; there was no significant relationship between anaemia (red blood cell density immediately prior to treatment), a common measure of virulence in this system (Mackinnon and Read 1999; Spence et al. 2013; Pollitt et al. 2014) and parasite half-life during treatment. Furthermore, higher growth rates correlated with increased susceptibility during drug treatment and had no significant effect on the ability of parasites to recover post-treatment. Thus, we find no evidence that artemisinin treatment will select for increased virulence; indeed, it may do the opposite.

Parasite traits are shaped by complex selection pressures, which will vary between transmission regions. Standing variation in traits underlying the susceptibility of parasite genotypes to drug treatment could mean that some parasite populations are predisposed to develop high-level resistance (Beez et al. 2011). The speed at which resistance develops within different populations is however difficult to predict; there are likely to be multiple, and potentially opposing, factors shaping both the probability of *de novo* resistance events and the strength of selection for high-level resistance once it arises (Read et al. 2011; zur Wiesch et al. 2011). For example, naturally less susceptible parasite populations are likely to have a greater biomass from which resistant mutants could arise but may be shielded from strong selection pressure. Testing how intrinsic variation in susceptibility to antiparasitic drugs influences the speed at which high-level resistance evolves will be an interesting and important avenue for future research.

## Data archiving statement

Data for this study are available from the Dryad Digital Repository: http://doi.org/10.5061/dryad.v244v.

## Supporting Information

**Table S1.** Model details for statistical analysis. **Table S2.** Fixing the starting point for clearance curves.
