# Supplementary material for: Understanding genetic variation in in vivo tolerance to artesunate: implications for treatment efficacy and resistance monitoring
Source: Evol Appl. 2014 Aug 11;8(3):296–304. doi: 10.1111/eva.12194 (PMC4380923; doi:10.1111/eva.12194)
Supplement: Supplementary file 1 [file eva0008-0296-sd1.pdf]

Supplementary information

S1: Model details for statistical analysis

We followed model simplification by sequentially dropping the least significant term and comparing the change in deviance with and without the term to χ^2 distributions (for linear mixed effect models) or F distributions (general linear models) until the minimal adequate model was reached. The first non-significant terms are shown in the order they were dropped from the top of the table and those included in the minimal model are highlighted in bold. A table is provided for each of the results referred to in the main text.

Table S1.1: *Line and treatment effects on overall parasite dynamics*

Linear mixed effect model					
Response variable	Model term	Residual d.f.	d.f. for term	X^2	P value
Parasite density (log10)	**Parasite genotype**				NA
	**Treatment**				NA
	**Day (as.factor)**				NA
	**Genotype*treatment**				NA
	**Genotype*day**				NA
	**Day*treatment**				NA
	**Genotype*day*treatment**	**953**	**110**	**744.2**	**<0.0001**
Random effect	**Mouse ID**	**953**	**1**		
Error structure for temporal autocorrelation	**corAR1(form=~day|mouse.id)** Phi = 0.84	**953**	**1**	**980**	**<0.0001**

Table S1.2: *Differences between parasite genotypes in clearance rates in treated infections*

General linear model with Gaussian error structure						
Response variable	Model term	Residual d.f.	d.f. for term	relationship	F	P value
Parasite half-life (log10)	Red blood cell density (day 6)	14	1		0.09	0.77
	Red blood cell density (day 11)	15	1		0.16	0.70
	Lag in clearance	16	1		1.04	0.32
	Pre-treatment growth	17	1		1.25	0.27
	**Parasite genotype**	**18**	**4**		**10.75**	**<0.0005**
	**Density of day 6**	**18**	**1**	**Negative**	**5.58**	**0.030**
Tukey post-hoc test for multiple comparisons of means						
Comparison				estimate	Z	p value
**AQ - AJ**				**+ 0.21**	**5.91**	**<0.001**
AS (pryR) - AJ					1.88	0.32
AS (wt) – AJ					2.49	0.09
**ER – AJ**				**+ 0.17**	**4.74**	**<0.001**
**AS (pryR) - AQ**				**- 0.13**	**3.53**	**<0.005**
**AS (wt) – AQ**				**- 0.11**	**3.32**	**0.008**
ER – AQ					1.18	0.76
AS (wt) – AS (pryR)					0.50	0.99
ER – AS (pryR)					2.02	0.25
ER – AS (wt)					1.82	0.36

Table S1.3: *Correlations between clearance rates and other infection parameters in treated infections*

General linear model with Gaussian error structure						
Response variable	Model term	Residual d.f.	d.f. for term	relationship	F	P value
Parasite half-life (log10)	Red blood cell density (day 11)	18	1		<0.001	0.99
	Red blood cell density (day 6)	19	1		0.43	0.52
	Lag in clearance	20	1		0.77	0.39
	Density on day 6	21	1		1.65	0.21
	**Intrinsic growth rate**	**22**	**1**	**Negative**	**15.55**	**<0.001**

Table S1.4: *Differences between parasite genotypes in clearance rates in untreated infections*

General linear model with Gaussian error structure						
Response variable	Model term	Residual d.f.	d.f. for term	relationship	F	P value
Parasite half-life (log10)	Red blood cell density (day 6)	16	1		0.05	0.82
	Pre-peak growth rate	17	1		2.13	0.16
	**Parasite genotype**	**18**	**4**		**6.48**	**<0.005**
	**Density of day 6**	**18**	**1**	**Negative**	**5.11**	**0.036**

Table S1.5: *Relative contribution of deteriorating host environment and drugs. Data from treated and untreated infections.*

General linear model with Gaussian error structure						
Response variable	Model term	Residual d.f.	d.f. for term	relationship	F	P value
Parasite half-life (log10)	Red blood cell density (day 6)	35	1		0.04	0.85
	**Pre-peak growth rate**	**36**	**1**	**Negative**	**4.15**	**0.049**
	**Density of day 6**	**36**	**1**	**Negative**	**9.48**	**<0.005**
	**Parasite genotype**					
	**Treatment**					
	**Parasite genotype * Treatment**	**36**	**4**		**9.10**	**<0.0001**

Table S1.6: *Parasite dynamics post treatment*

Linear mixed effect model					
Response variable	Model term	Residual d.f.	d.f. for term	X^2	P value
Parasite density (log10)	Lag in clearance	243	1	0.004	0.95
	Growth rate pre-treatment	244	1	0.18	0.68
	Half-life during treatment	245	1	0.13	0.72
	Density at end of treatment	246	1	1.44	0.23
	**Parasite genotype**				
	**Day**				
	**Parasite genotype * Day**	**247**	**52**	**160.5**	**<0.0001**
Random effect	**Mouse ID**	**247**	**1**		
Error structure for temporal autocorrelation	**corAR1(form=~day|mouse.id)**<br>**Phi = 0.78**	**247**	**1**	**327.5**	**<0.0001**

Table S1.7: *The effect of parasite genotype on cumulative parasite density post-treatment*

General linear model with Gaussian error structure						
Response variable	Model term	Residual d.f.	d.f. for term	relationship	F	P value
Cumulative density (log10)	End of treatment density	15	1		0.48	0.50
	Parasite half life	16	1		0.94	0.35
	Growth rate pre-treatment	17	1		1.12	0.30
	End of treatment RBC density	18	1		3.54	0.076
	**Parasite genotype**	**19**	**4**		**9.74**	**<0.0005**
Tukey post-hoc test for multiple comparisons of means						
Comparison				estimate	Z	p value
**AQ - AJ**				**+ 1.35**	**3.94**	**<0.001**
AS (pryR) - AJ					0.75	0.94
**AS (wt) – AJ**				**+ 0.95**	**2.78**	**0.043**
**ER – AJ**				**+ 1.19**	**3.47**	**0.005**
**AS (pryR) - AQ**				**- 1.60**	**4.98**	**<0.002**
AS (wt) – AQ					1.23	0.73
ER – AQ					0.50	0.98
**AS (wt) – AS (pryR)**				**+ 1.21**	**3.75**	**0.002**
**ER – AS (pryR)**				**+ 1.44**	**4.48**	**<0.001**
ER – AS (wt)					0.73	0.95

Table S1.8: *Correlations between cumulative density post-treatment and other infection parameters*

General linear model with Gaussian error structure						
Response variable	Model term	Residual d.f.	d.f. for term	relationship	F	P value
Cumulative density (log10)	Growth rate pre-treatment	19	1		0.35	0.77
	Parasite half-life	20	1		0.29	0.60
	End of treatment RBC density	21	1		2.95	0.10
	**End of treatment density**	**22**	**1**	**Positive**	**7.50**	**0.012**

Table S1.9: *Line and treatment effects on infection virulence: Whole infection*

Linear mixed effect model: RBC					
Response variable	Model term	Residual d.f.	d.f. for term	X^2	P value
Red blood cell density (relative to baseline pre-infection)	Parasite genotype*treatment	14	4	2.90	0.58
	Infection growth rate (days 3-6)	18	1	0.097	0.76
	Parasite genotype	19	4	9.18	0.057
	**Treatment**	**23**	**1**	**5.41**	**0.02**
	**Day**		**22**	**690.8**	**<0.0001**
Random effect	**Mouse ID**	**23**	**1**		
Error structure for temporal autocorrelation	**corAR1(form=~day|mouse.id)** **Phi = 0.77**	**23**	**1**	**963.6**	**<0.0001**
Linear mixed effect model: Weight					
Mouse weight (relative to baseline pre-infection)	Parasite genotype*treatment	14	4	3.66	0.45
	Parasite genotype	18	4	3.055	0.54
	Infection growth rate (days 3-6)	22	1	0.50	0.48
	**Treatment**	**23**	**1**	**18.10**	**<0.0001**
	**Day**	**23**	**22**	**301.25**	**<0.0001**
Random effect	**Mouse ID**	**23**	**1**		
Error structure for temporal autocorrelation	**corAR1(form=~day|mouse.id)** **Phi = 0.74**	**23**	**1**	**758.4**	**<0.0001**

Table S1.10: *Line and treatment effects on infection virulence: Initial acute infection (days 7-17)*

Linear mixed effect model: RBC					
Response variable	Model term	Residual d.f.	d.f. for term	X^2	P value
Red blood cell density (relative to baseline pre-infection)	**Parasite genotype**		**4**		
	**Treatment**		**1**		
	**Parasite genotype*treatment**	**27**	**4**	**16.78**	**0.002**
	**Day**	**27**	**9**		**<0.0001**
Random effect	**Mouse ID**	**27**	**1**		
Error structure for temporal autocorrelation	**corAR1(form=~day|mouse.id)** **Phi = 0.64**	**27**	**1**	**159.8**	**<0.0001**
Linear mixed effect model: Weight					
Mouse weight (relative to baseline pre-infection)	Parasite genotype*treatment	27	4	4.00	0.41
	Parasite genotype	31	4	5.10	0.28
	**Treatment**	**32**	**1**	**27.38**	**<0.0001**
	**Day**	**32**	**9**	**186.69**	**<0.0001**
Random effect	**Mouse ID**	**32**	**1**		
Error structure for temporal autocorrelation	**corAR1(form=~day|mouse.id)** **Phi = 0.76**	**32**	**1**	**313.23**	**<0.0001**

Table S1.11: *Line and treatment effects on infection virulence: Late infection (days 18-26)*

Linear mixed effect model: RBC					
Response variable	Model term	Residual d.f.	d.f. for term	X^2	P value
Red blood cell density	**Parasite genotype**		**4**		
(relative to baseline pre-infection)	**Treatment**		**1**		
	**Parasite genotype*treatment**	**27**	**4**	**11.07**	**0.026**
	**Day**	**27**	**7**	**55.34**	**<0.0001**
Random effect	**Mouse ID**	**27**	**1**		
Error structure for temporal autocorrelation	**corAR1(form=~day|mouse.id)** **Phi = 0.56**	**27**	**1**	**118.34**	**<0.0001**
Linear mixed effect model: Weight					
Mouse weight	**Parasite genotype**		**4**		
(relative to baseline pre-infection)	**Treatment**		**1**		
	**Parasite genotype*treatment**	**27**	**4**	**10.12**	**0.038**
	**Day**	**27**	**7**	**14.38**	**0.045**
Random effect	**Mouse ID**	**27**	**1**		
Error structure for temporal autocorrelation	**corAR1(form=~day|mouse.id)** **Phi = 0.65**	**27**	**1**	**81.02**	**<0.0001**

S2: Fixing the starting point for clearance curves

As we only took parasite density measurements every 24hrs (once per cell cycle) it is possible the peak could have occurred between measurements. In order to ensure that our choice of start point for the clearance curves did not influence whether we found variation in clearance rates between the parasite genotpes we re-fitted curves with a fixed start point on day 6 (the start of treatment). This led to a slightly worse fit to the data (mean $R^2 = 0.91 \pm 0.02$ S.E.) but did not effect the conclusions of our analysis (S1).

Table S2: *Analysis of genetic variation in clearance rate when all clearance curves fitted from day 6*

General linear model with Gaussian error structure						
Response variable	Model term	Residual d.f.	d.f. for term	relationship	F	P value
Parasite half-life (log10)	Red blood cell density (day 6)	14	1		0.19	0.67
	Lag in clearance	16	1		0.37	0.55
	Red blood cell density (day 11)	15	1		0.67	0.42
	Pre-treatment growth	17	1		2.19	0.16
	**Density of day 6**	**18**	**1**	**Negative**	**5.22**	**0.035**
	**Parasite genotype**	**18**	**4**		**7.19**	**0.0012**
Tukey post-hoc test for multiple comparisons of means						
Comparison				estimate	Z	p value
**AQ - AJ**				**+ 0.16**	**4.82**	**<0.001**
AS (pryR) - AJ					0.96	0.87
AS (wt) – AJ					1.92	0.30
**ER – AJ**				**+ 0.11**	**3.23**	**0.011**
**AS (pryR) - AQ**				**- 0.12**	**3.53**	**0.004**
**AS (wt) – AQ**				**- 0.09**	**2.84**	**0.036**
ER – AQ					1.60	0.49
AS (wt) – AS (pryR)					0.99	0.86
ER – AS (pryR)					1.68	0.44
ER – AS (wt)					1.03	0.84
